# Piezoelectric Versus Conventional Rotary Techniques for Impacted Third Molar Extraction: A Meta-analysis of Randomized Controlled Trials

**DOI:** 10.1097/MD.0000000000001685

**Published:** 2015-10-16

**Authors:** Qian Jiang, Yating Qiu, Chi Yang, Jingyun Yang, Minjie Chen, Zhiyuan Zhang

**Affiliations:** From the Department of Oral and Maxillofacial Surgery, Shanghai Ninth People's Hospital, Shanghai Jiaotong University School of Medicine, Shanghai, China (QJ, YQ, CY, MC, ZZ); Rush Alzheimer's Disease Center (JY); and Department of Neurological Sciences, Rush University Medical Center, Chicago, Illinois (JY).

## Abstract

Impacted third molars are frequently encountered in clinical work. Surgical removal of impacted third molars is often required to prevent clinical symptoms. Traditional rotary cutting instruments are potentially injurious, and piezosurgery, as a new osteotomy technique, has been introduced in oral and maxillofacial surgery. No consistent conclusion has been reached regarding whether this new technique is associated with fewer or less severe postoperative sequelae after third molar extraction.

The aim of this study was to compare piezosurgery with rotary osteotomy techniques, with regard to surgery time and the severity of postoperative sequelae, including pain, swelling, and trismus.

We conducted a systematic literature search in the Cochrane Library, PubMed, Embase, and Google Scholar.

The eligibility criteria of this study included the following: the patients were clearly diagnosed as having impacted mandibular third molars; the patients underwent piezosurgery osteotomy, and in the control group rotary osteotomy techniques, for removing impacted third molars; the outcomes of interest include surgery time, trismus, swelling or pain; the studies are randomized controlled trials.

We used random-effects models to calculate the difference in the outcomes, and the corresponding 95% confidence interval. We calculated the weighted mean difference if the trials used the same measurement, and a standardized mean difference if otherwise.

A total of seven studies met the eligibility criteria and were included in our analysis. Compared with rotary osteotomy, patients undergoing piezosurgery experienced longer surgery time (mean difference 4.13 minutes, 95% confidence interval 2.75–5.52, *P* < 0.0001). Patients receiving the piezoelectric technique had less swelling at postoperative days 1, 3, 5, and 7 (all *P*s ≤0.023). Additionally, there was a trend of less postoperative pain and trismus in the piezosurgery groups.

The number of included randomized controlled trials and the sample size of each trial were relatively small, double blinding was not possible, and cost analysis was unavailable due to a lack of data.

Our meta-analysis indicates that although patients undergoing piezosurgery experienced longer surgery time, they had less postoperative swelling, indicating that piezosurgery is a promising alternative technique for extraction of impacted third molars.

## Abstract 2

Supplemental Digital Content is available in the text

## INTRODUCTION

Impacted third molars are frequently encountered in clinical work, with a prevalence of 33% to 58.7%.^1–4^ It has been well documented that impacted third molars, either partial or complete, are associated with several complications, including pericoronitis, regional pain, odontogenic abscesses, trismus, distal caries, cysts, tumors, and arch crowding.^5–8^ Therefore, symptomatic or asymptomatic impacted third molars are often extracted to reduce the above-mentioned clinical symptoms.

The surgical removal of impacted third molars may lead to various postoperative side effects, including pain, swelling, trismus, nerve injury, bleeding, and dry sockets.^9,10^ Different strategies are adopted to reduce these complications, including changing the technique of the osteotomy.^11^

Traditionally, impacted third molars are often removed using rotary osteotomy techniques. However, conventional rotary cutting instruments are potentially injurious because they can generate excessively high temperatures during bone drilling, which leads to marginal osteonecrosis, and can impair osseous regeneration and healing.^12–14^ Recently, along with the tendency toward minimally invasive surgery, piezosurgery—a new osteotomy technique—has been introduced in oral and maxillofacial surgery. Using a piezoelectric device, piezosurgery has a much lower risk of visible injury to the adjacent soft tissues, leading to more favorable osseous repair and remodeling.^13^ Randomized controlled trials (RCTs) have been conducted to compare piezosurgery with traditional rotary techniques regarding postoperative sequelae after third molar extraction, with inconsistent results reported.^15–21^

Therefore, in this study, we performed an extensive literature search of RCTs and conducted meta-analyses to compare piezosurgery with conventional rotary osteotomy techniques, with regard to surgery time and postoperative sequelae, including pain, swelling, and trismus.

## METHODS

### Eligibility Criteria

The following inclusion criteria were used to determine study eligibility: the patients were clearly diagnosed as having impacted mandibular third molars; the patients underwent piezosurgery osteotomy techniques, and in the control group rotary osteotomy techniques, for removing the impacted third molars; the outcomes of interest included surgery time, trismus, swelling, or pain, as assessed using the visual analog scale (VAS); and the studies were RCTs. Ethical approval was not necessary, as this study was based on published data.

### Search Strategy

Two authors (QJ and JY) performed an extensive literature search in the Cochrane Library, PubMed, Embase, and Google Scholar for papers published up to December 23, 2014. The keywords used in the literature search can be found in the supplementary file.

We retrieved all potential relevant publications, which were evaluated for inclusion in this study. We also searched for additional studies that might be missed by the database search by manually searching the reference list for all relevant publications. The two authors performed the literature search independently, and any disagreement was resolved by a group discussion.

### Data Extraction

The following data were extracted and recorded independently by the two reviewers (QJ and YQ), following a prespecified protocol: first author's name, year of publication, country of origin, hospital name, study design, mean age of participants, sample size, impacted type, duration of surgery time, postoperative pain, swelling, and trismus. Any disagreement or lack of clarity was resolved through a group discussion. If a trial reported data through a figure, Engauge Digitizer version 4.1 (http://digitizer.sourceforge.net/) was used to read the data and efforts were made to contact the authors if additional data were needed. An assessment of study validity was done using Cochrane Collaboration's tool, which is one of the most popular tools for assessing the risk of bias for RCTs.^22^ It is composed of the following six dimensions: random sequence generation; allocation concealment; blinding; addressing of incomplete outcome data; selective outcome reporting; and other apparent risks of bias.

### Data Analysis

Random-effects models were used to calculate the difference in the outcomes and the corresponding 95% confidence intervals (CIs). Forest plots were used to represent the pooled mean differences and 95% CIs. If the trials used the same measurement instrument, the weighted mean difference (WMD) and its 95% CIs were calculated. Otherwise, the standardized mean differences (SMDs) and their 95% CIs were calculated. We first conducted our meta-analyses by study design (parallel study and split-mouth design), and then pooled the analysis results for both designs. *I*^2^ was used to assess the between-study heterogeneity, and a *P* value <0.20 was considered to indicate statistically significant heterogeneity among the studies.

This study was reported according to the PRISMA guidelines.^23^ All statistical analyses were performed using Stata 11.2 (StataCorp LP, College Station, TX), and a *P* value <0.05 was considered to be statistically significant.

## RESULTS

### Study Selection and Characteristics

**FIGURE 1:**
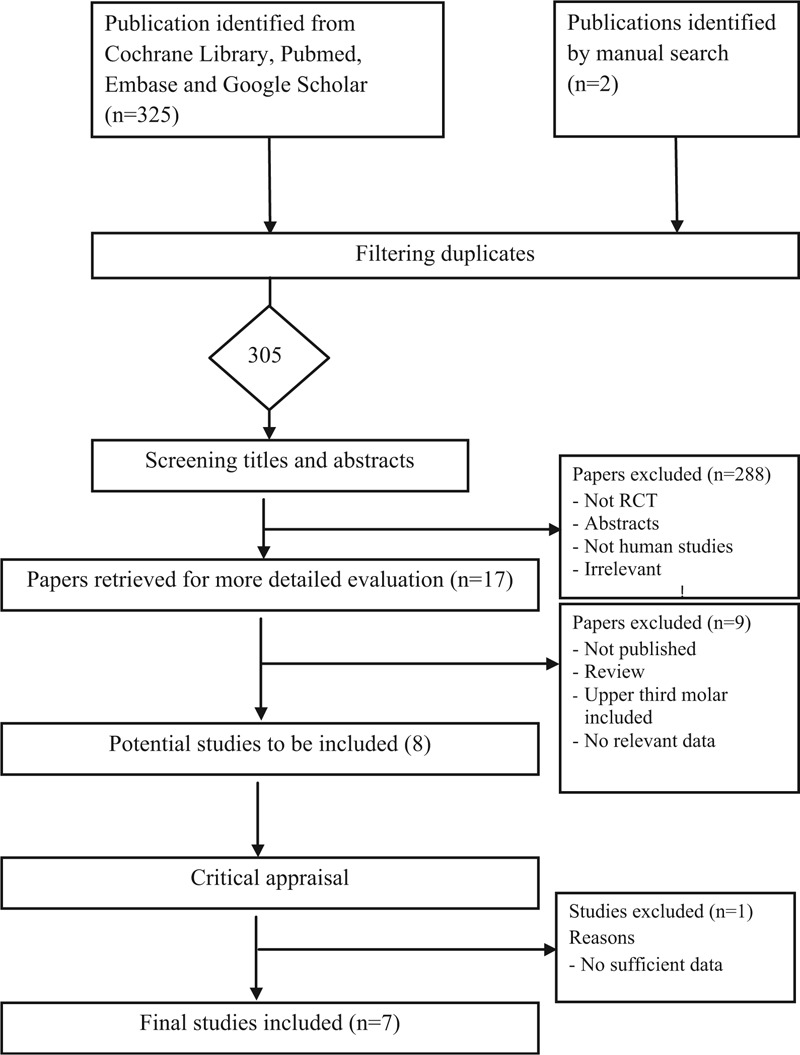
Flow diagram of the literature search and selection process. Please refer to the “Methods” section for more details.

The selection of eligible studies included in the meta-analyses is presented in Figure 1. Following our predefined search strategy, our initial search identified 305 potential publications. We excluded 288 publications because they were not RCTs, not about humans, or because they were published abstracts or were irrelevant, leading to 17 studies which were retrieved for more detailed evaluations. Nine additional studies were excluded because they were not published, were reviews, included the upper third molar, or there were no sufficient data. This led to 8 potentially relevant publications to be included in our meta-analysis. Finally, we further excluded 1 more study because there were insufficient data despite efforts to contact the authors.^24^ As a result, a total of 7 studies met the eligibility criteria and were included in our meta-analysis. These 7 eligible studies were published from 2008 through 2014.^15–21^ Among them, 3 used a parallel-group design (each patient as a study unit),^15,16,18^ and 4 used a split-mouth design (each tooth as a study unit).^17,19–21^ Antibiotics were prescribed in all studies. Table 1 summarizes the characteristics of the 7 included studies.

**TABLE 1:**
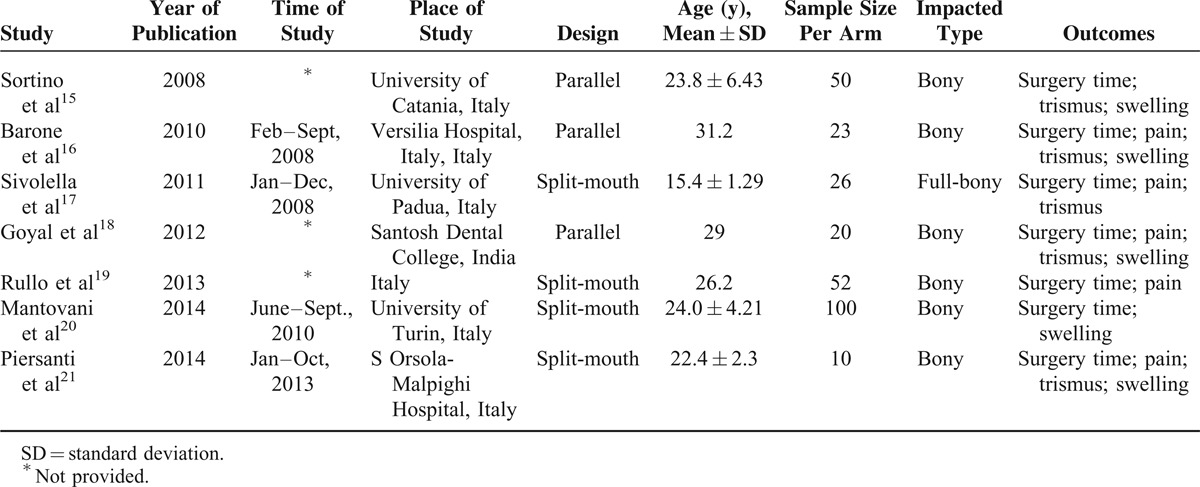
Characteristics of Included Studies

### Risk of Biases in Included Studies

**FIGURE 2:**
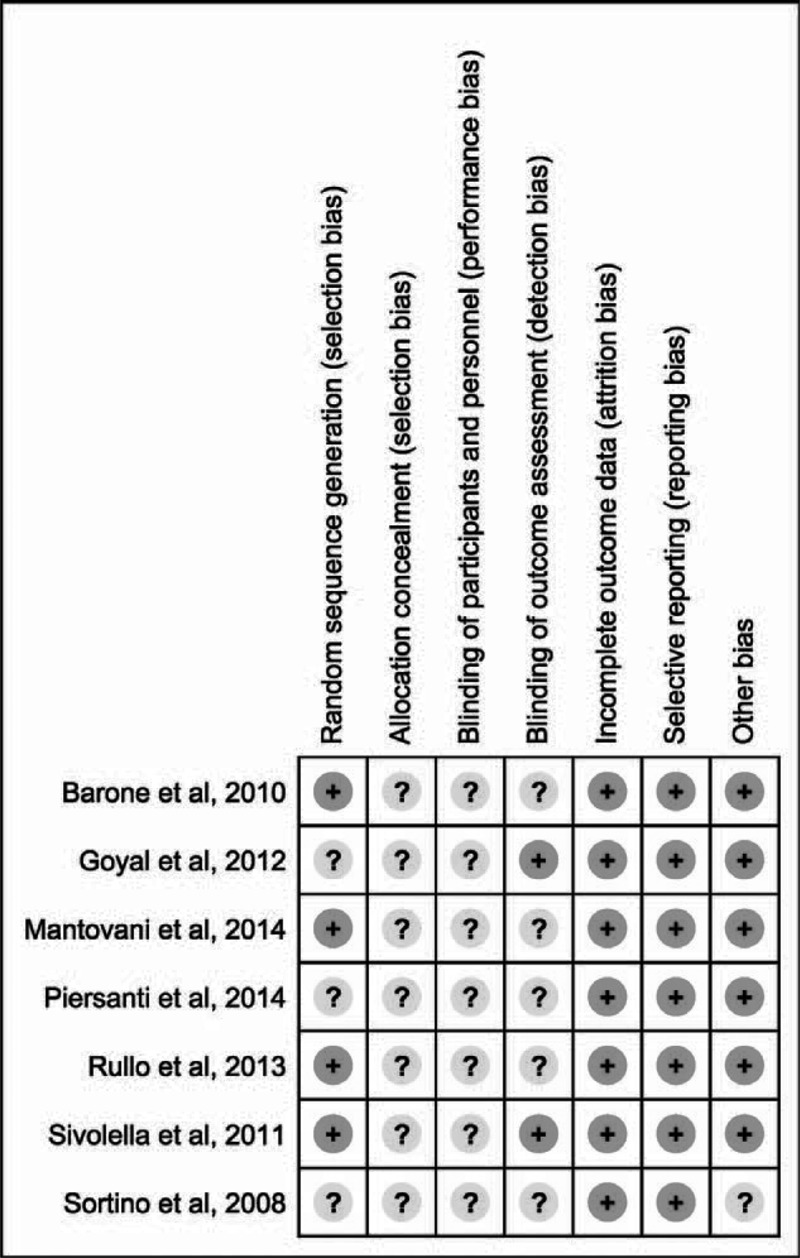
Assessment of risk of bias of included studies.

Figure 2 summarizes the assessment of the risk of bias of the included studies. Because double blinding was not possible, all of the studies were judged as having an unclear risk of performance bias. Two studies reported that the patients were allocated into the piezosurgery and rotary groups according to a computer-generated randomization list,^16,17^ 1 study used a table of random numbers,^20^ and another study used a coin toss.^19^ These 4 studies were considered to be at low risk and the other 3 studies were considered to be at an unclear risk in the random sequence generation.

### Surgery Time

**FIGURE 3:**
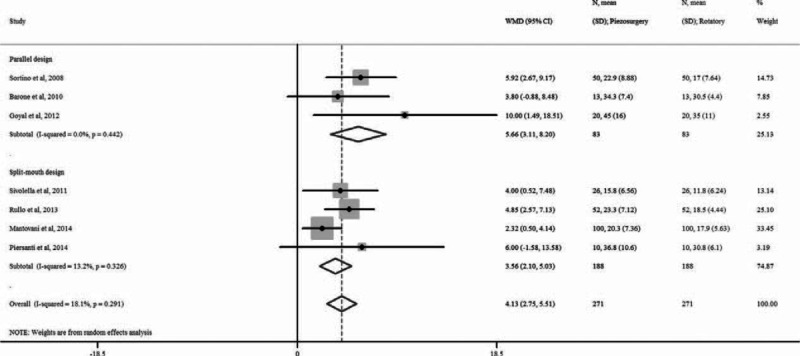
Forest plot for meta-analysis of the difference in surgery time between piezosurgery versus rotary osteotomy technique in third molar extraction.

All of the trials evaluated the surgery time, and indicated longer average surgery time in the piezosurgery group than in the rotary group (eTable 1, http://links.lww.com/MD/A449). The meta-analysis indicated significantly longer surgery time in the piezosurgery group, compared with the rotary group (WMD 4.13 minutes, 95% CI 2.75–5.52, *P* < 0.0001) (Figure 3). The subgroup analysis by study design revealed significantly longer surgery time in the piezosurgery group for both the parallel design and the split-mouth design studies. There was low heterogeneity among the included studies (all *I*^*2*^ < 19%).

### Pain

**FIGURE 4:**
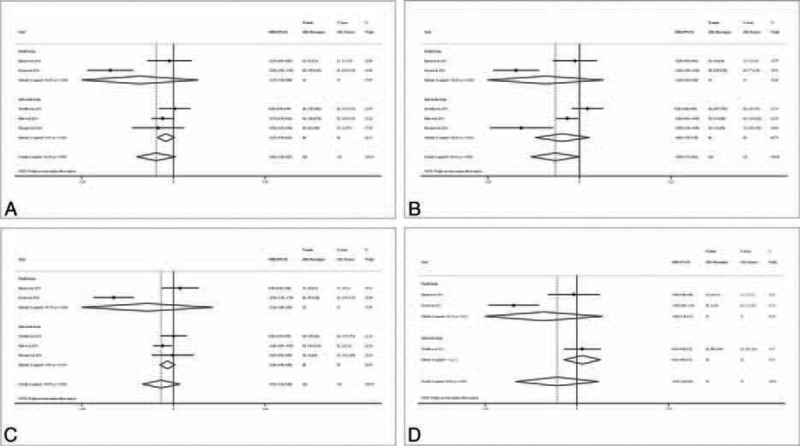
Forest plot for meta-analysis of the difference in postoperative pain between piezosurgery versus rotary osteotomy technique in third molar extraction. A, Day 1 after surgery; B, day 3 after surgery; C, day 5 after surgery; D, day 7 after surgery.

With the exception of 1 trial,^15^ all of the trials reported pain scores using the VAS (10-unit or 100-unit) on different postoperative days (eTable 2, http://links.lww.com/MD/A449). Furthermore, the postoperative pain decreased following the third molar extraction for both groups. The meta-analysis indicated that there was no statistically significant difference in pain between the piezosurgery group and the rotary group on any of the postoperative days (Figure 4). However, in the first few days after surgery, there was a trend of less pain in the piezosurgery group than in the rotator group; the difference is nominal, but not statistically significant (day 1: SMD −0.61, 95% CI −1.29 to 0.07, *P* = 0.078; day 3: SMD −0.85, 95% CI −1.71 to 0.006, *P* = 0.052).

### Trismus

**FIGURE 5:**
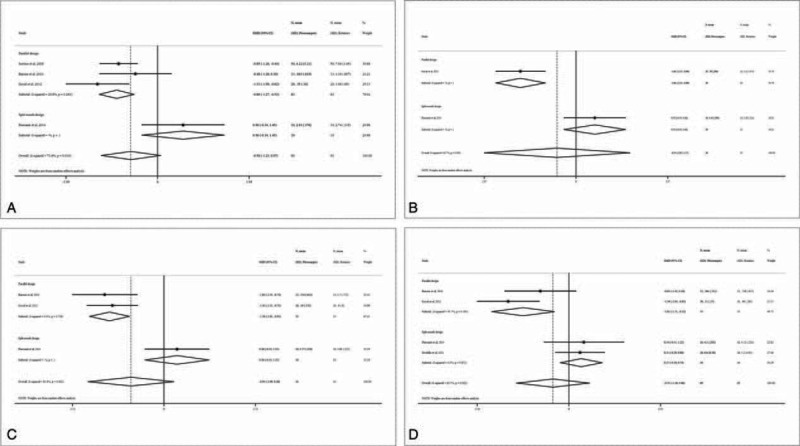
Forest plot for meta-analysis of the difference in postoperative trismus between piezosurgery versus rotary osteotomy technique in third molar extraction. A, Day 1 after surgery; B, day 3 after surgery; C, day 5 after surgery; D, day 7 after surgery.

Five studies evaluated postoperative trismus, all of which measured the maximum mouth opening at specific time points (eTable 3, http://links.lww.com/MD/A449).^15–18,21^ The meta-analysis did not indicate a significant difference in trismus between the piezosurgery group and the rotary group on any of the postoperative days (Figure 5). However, a subgroup analysis indicated a statistically significant decrease in trismus on all of the postoperative days (1, 3, 5, and 7) in the piezosurgery group in studies with a parallel design, but not in studies with a split-mouth design, probably due to the small sample size.

### Swelling

**FIGURE 6:**
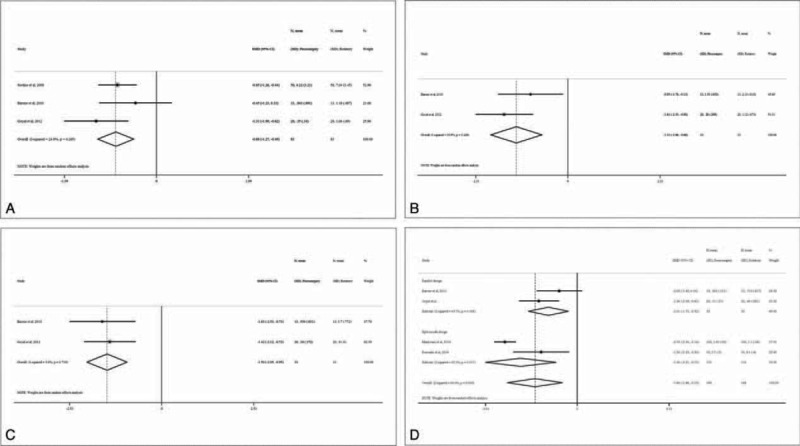
Forest plot for meta-analysis of the difference in postoperative swelling between piezosurgery versus rotary osteotomy technique in third molar extraction. A, Day 1 after surgery; B, day 3 after surgery; C, day 5 after surgery; D, day 7 after surgery.

Five studies reported swelling at specific time points, but used different measurements (eTable 4, http://links.lww.com/MD/A449).^15,16,18,20,21^ The meta-analysis indicated that patients in the piezosurgery group had significantly reduced facial swelling than those in the rotary group on all postoperative days (all *P*s ≤0.023; Figure 6). A subgroup analysis by study design is only available for postoperative day 7, which indicated a significant difference for both the parallel design and the split-mouth design studies.

## DISCUSSION

In this study, we performed a systematic literature search and conducted meta-analysis to compare piezosurgery and conventional rotary osteotomy techniques in third molar extraction. We found that although the patients undergoing piezosurgery experienced longer surgery time, they developed less swelling when compared with those undergoing conventional rotary techniques. Patients who underwent piezosurgery also seemed to have experienced less pain during piezosurgery, and developed less postoperative trismus, although these findings are not conclusive due to the limited sample size. To the best of our knowledge, this is the first meta-analysis of RCTs to compare piezosurgery with rotary osteotomy techniques in third molar extraction.

Compared with surgery using rotary techniques, piezosurgery was more time-consuming due to the slower micrometric cutting action of the piezoelectric device. Surgery time using the ultrasonic osteotomy tended to be shorter as the surgeons accumulated more experience.^25^ Therefore, although the piezoelectric technique is associated with longer surgery time, we believe that with increased experience and the improvement of the technique, piezosurgery will witness reduced surgery time.

Although there was a trend of less pain in piezosurgery, the difference did not reach statistical significance; however, we may not have sufficient power due to the limited sample size. In addition, the validity of the results might be affected by several factors. Piezosurgery and high-speed air turbine hand pieces are not the same across studies. The impacted types of the mandibular third molars also differ among trials. Moreover, the surgeons’ skills and experiences and patients’ pain sensitivity might be different, which could influence the assessment of the level of postoperative pain. More or larger homogeneous RCTs are needed to validate our findings.

In addition to less swelling, and possibly less trismus, piezosurgery has other advantages. The bone samples harvested suing piezosurgery were characterized by the integrity of the bony structure, a well defined osteotomy, but no evidence of bone heat osteonecrosis.^19^ Other clinical trials of the maxillary sinus floor elevation also confirmed a lack of coagulative necrosis on the surfaces of the bony segments via ultrasound osteotomy.^26,27^ Research on the osseointegration of oral titanium implants reported that piezosurgery induced an earlier increase in bone morphogenic proteins (BMPs), controlled the inflammatory process better, and stimulated bone remodeling.^10,28^ These data suggest that the alveolar bone removed by the use of a piezoelectric device for third molar extraction can be used for bone augmentation in implant placement.

Our study has some limitations. First, the number of included RCTs and the sample size of each trial were relatively small. Second, double blinding was not possible for obvious reasons, leading to unclear performance bias. Third, piezoelectric devices generally cost more than rotary devices; however, a cost analysis was unavailable in this meta-analysis due to the lack of data. Moreover, data are scarce on other postoperative complications after the extraction of impacted mandibular third molars, such as alveolitis, infection, and paresthesia; therefore, a meta-analysis of such complications was not feasible. Future studies are needed to compare the incidence of other common postoperative complications between the two approaches.

In summary, in this study, we conducted an extensive literature search and performed meta-analysis to compare piezosurgery with rotary osteotomy techniques in third molar extraction. We found that the patients undergoing piezosurgery had significantly less swelling. There was also a trend of less pain and trismus in piezosurgery, when compared with rotary osteotomy. More large-scale and multicenter RCTs using a unified grading system and evaluation index are needed to validate our findings and provide guidance for clinical applications.
